# *Helicobacter pylori* neutrophil-activating protein: a potential Treg modulator suppressing allergic asthma?

**DOI:** 10.3389/fmicb.2015.00493

**Published:** 2015-06-01

**Authors:** Anjna Sehrawat, Siddharth Sinha, Abhishek Saxena

**Affiliations:** Department of Biotechnology, TERI UniversityNew Delhi, India

**Keywords:** *Helicobacter pylori*, allergic asthma, neutrophil-activating protein, hygiene hypothesis, Tregs, molecular docking

## Abstract

The ultimate aim of the immune system is to eliminate pathogens without being harmful to the host. But what if eliminating the pathogen in itself is discomforting for the host? One such emerging case is of *Helicobacter pylori*. Modern medicine, infantile vaccination, and ultra-hygienic conditions have led to progressive disappearance of *H. pylori* in different parts of the world. However, the adversities caused by *H. pylori’s* absence are much larger than those caused by its presence. Asthma is rising as an epidemic in last few decades and several reports suggest an inverse-relationship between *H. pylori’s* persistence and early-life onset asthma. Regulatory T cells play an important role in both the cases. This is further supported by experiments on mouse-models. Hence, need of the hour is to discern the relationship between *H. pylori* and its host and eliminating its negative impacts without disturbing our indigenous microbiota. To resolve whether *H. pylori* is a pathogen or an amphibiont is another important side. This review explores the biological basis of *H. pylori*-induced priming of immune system offering resistance to childhood-onset asthma. HP-NAP–Tregs interaction has been predicted using molecular docking and dynamic simulation.

## Introduction

Ever since it was proposed that a reduced exposure to infectious diseases during childhood was correlated with the development of allergies later in life (Strachan, 1989), the so-called *hygiene hypothesis* has become a topic for active research. With time, the hypothesis has grown in two opposite directions as far as complexity of the problem is concerned. In its most reductionist form, especially in popular discourse, it is reduced to the notion that an aseptic ‘western’ lifestyle leads to a higher incidence and earlier development of allergies. The counterpoint is that the situation is much more complex, and a more accurate statement would be that “using antibiotics, antibacterial soaps, and clean environments cause increased risk of allergies” (Graham-Rowe, 2011). Even the term “hygiene hypothesis” can be misleading, seemingly reducing a complicated problem into a simple “dirty is healthy” prescription. Numerous studies have hypothesized that allergies are linked to environment, education, family size, and economic status. As living conditions have improved (both in the developed nations and the developing nations), drastic changes have occurred in lifestyles and standards of living, which have distanced humans from their “old friends”: microorganisms and helminths that once lived in the body and conferred protection from atopic allergies (the old-friends hypothesis; Rook et al., 2013).

*Helicobacter pylori*, a Gram-negative bacterium infecting nearly half the world’s population, has been viewed mostly as a chronic pathogen that causes peptic and duodenal ulcers since the pioneering work of Marshall and Warren (1984). Additionally, it is found to be a strong predisposing factor for gastric cancer and classified as a Class I carcinogen by the World Health Organization (IARC Working Group on the Evaluation of Carcinogenic Risks to Humans, 1994). However, *H. pylori* may be involved in a more complicated interaction with the human immune system, given its ability to persist throughout the lifetime of the host. The balance achieved by *H. pylori* in stimulating the host’s immune system sufficiently to cause inflammation and at the same time modulating the host immune response to prevent its own elimination, is worth mentioning. Studies have revealed that the association of *H. pylori* with humans is ancient (≥60,000 years) dating back to the migration out of Africa and subsequent spread to the rest of the world (Covacci et al., 1999). Historically recent improvements in sanitation and healthcare have led to a lower incidence of *H. pylori* in human populations (Bruce and Maaroos, 2008). This means that developed nations exhibit a progressively declining incidence of *H. pylori* compared to developing nations, but there is also an overall decline in *H. pylori* prevalence over time regardless of the economic status of the nation as a whole. As an example of the latter case, an ELISA-based study conducted in Guangzhou City (China) found that 49.3% of the tested individuals were positive for *H. pylori* in 2003 as opposed to 62.5% in a similar study conducted in 1993 (Chen et al., 2007). Similar studies on *H. pylori* prevalence in Canada have linked increased seropositivity to *H. pylori* with lower educational status of the individuals (Naja et al., 2007). A study by Ahmed et al. (2007), found that a higher prevalence of *H. pylori* correlated with a lower household hygiene and limited access to potable water. In this article, we propose a mechanism whereby decreasing incidence of *H. pylori* infection could contribute to the increasing incidence of one allergic condition, viz., asthma.

Asthma is a chronic inflammatory disorder of the airways that manifests as a narrowing of the airways and consequent breathlessness, and is symptomatically treated by aerosolized bronchodilators. Allergy, especially to airborne allergens, may also result in ‘allergic asthma.’ A worrisome yet intriguing trend is that there is a higher incidence of allergies in populations (especially children) that are less exposed to *H. pylori* colonization and seem to be at a higher risk of allergic rhinitis and asthma due to the cohort effect. The inverse link between childhood onset of asthma and *H. pylori* colonization (especially with *cagA^+^* strains) in western populations has been observed (Chen and Blaser, 2007; Blaser et al., 2008). The *H. pylori* cytotoxin associated gene A (CagA) has been implicated in peptic ulcer disease but has also been shown to play a role in preventing the development of allergies in the host (Boeing, 2012).

## Epidemiological Evidence

In one of the most comprehensive studies conducted for understanding the relationship between *H. pylori* infection and the incidence of asthma in developed populations, Chen and Blaser (2007) have analyzed data from the Third National Health and Nutrition Examination Survey of the U.S. (NHANES III; October 1988–October 1994). An overall pattern of association of asthma and *H. pylori* infection was not visible in the study, there; however, was an age-related trend in the prevalence of *cagA^+^ H. pylori* strains and current asthma, with a pronounced inverse relationship in younger participants (Chen and Blaser, 2007). In 2008, the same group followed up the above-mentioned analysis with another one, on data obtained from the first part of NHANES IV (1999–2000). This study was aimed at testing the hypothesis that *H. pylori* infection and onset of asthma and other allergies had an inverse relationship only in children and young respondents (the median age being 25 years), as compared to 43 years of the earlier mentioned NHANES III study (Chen and Blaser, 2008). The study established an inverse relationship between *H. pylori* infection in children and onset of asthma during early years. The authors explain this observation by providing the fact that increased intake of antibiotics during early years by children may result in decreased colonization by *H. pylori*. In fact, the authors report that 11.3% of respondents who were ≤10 years of age had taken antibiotics 1 month before the study. The seroprevalence of *H. pylori* has been reduced to <10% in such cases of children native to the U.S. and other industrialized countries.

In yet another study, Reibman et al. (2008) report a similar relationship between presence of H. pylori and incidence of asthma and other allergies, although the study does not find any inverse relationship between H. pylori prevalence and immunoglobulin E (IgE) concentration in the serum. This result points to the fact that there might be other mechanisms independent of IgE mediated ones that the bacterium uses for modifying asthma status. Possible mechanisms may involve immune modification of the host system (Reibman et al., 2008). Another such study, conducted by Hugg et al. (2008) have made a comparison between Finnish and West Russian school children to determine their susceptibility to various kinds of allergic diseases.

Although some studies (for instance Thirumurthi and Graham, 2012) argue that the link between *H. pylori* prevalence and allergies is not real and appears due to misunderstanding of the concept of protection (Thirumurthi and Graham, 2012), most studies have found overwhelming evidence of the inverse relationship between allergies, asthma, and *H. pylori* (Chen and Blaser, 2007, 2008; Atherton and Blaser, 2009).

## Regulatory T Cells: Connecting Link between Asthma and *Helicobacter pylori*

Regulatory T cells (hereafter referred to as Tregs), a “self-check” built into the immune system to suppress immune responses of other cells are one of the recently discovered components of the immune system. As per the “self/non-self hypothesis,” all foreign matter is treated as potentially dangerous by the immune system, which may compromise on the beneficial effects of commensals and symbionts. Therefore such associations between host and other organisms can be explained by the formation of regulatory cells (Dembic, 2008). Tregs might function by translating the presence of foreign organism into a “net profit” message, in the case of a beneficial association with the host. Reports also provide examples of subversion of Tregs by pathogens. For example, parasitic infections like malaria (Adalid-Peralta et al., 2011), mycobacterial infections like tuberculosis (Arram et al., 2013), and retroviral infections like HIV result in increased Treg activity helping in their long-term survivals (Punkosdy et al., 2011).

While the precise understanding of the immunosuppressive mechanism of Tregs is still lacking and there is a need to investigate it further, *in vitro* experiments have suggested the requirement of cell contact mediated regulation involving secreted immunosuppressive factors such as interleukin 9 (IL-9), IL-10, transforming growth factor *beta* (TGF β), and other co-stimulatory factors such as GITR (glucocorticoid-induced TNFR family related gene) and CTLA-4 (cytotoxic T-lymphocyte-associated protein 4).

### Understanding the Immunology of Allergic Asthma

Till very recently, the Th2 (T-helper type 2) cells were implicated in the response to allergens and in the development of asthma (Cohn et al., 2004). Th1/Th2 imbalance was one of the first hypotheses to explain asthma immunology (Oertli et al., 2012). Development of allergic asthma has been attributed to the allergen-specific CD4^+^ T cells that are present in high frequency in the peripheral blood flow of the patient. These cells are characterized by the Th2 cytokines IL-4, IL-5, IL-9, and IL-13 which are responsible for allergic pathophysiology leading to the increased seropresence of IgE (Nauta et al., 2008).

Several studies conducted in mice as well as humans (reviewed in Ryanna et al., 2009) have established the role of Tregs in controlling acute and chronic asthma, thus making them popular targets of therapeutic importance (Ryanna et al., 2009). However, most research that has gone into effects of Tregs on asthma has utilized the Tregs from peripheral blood and has not assessed the function of Tregs in the respiratory pathway. Even within Tregs, the role of specific types, i.e., IL-10 secreting Tregs (that are more common in the lungs) and TGF-β secreting Tregs (more in the gut, but shown to play a role in asthma regulation) need to be studied in greater detail to establish a pathway and hence come up with viable solutions for treatment of asthma. Overall, however, it is accepted that CD4^+^ CD25^+^ Foxp3^+^ natural Tregs play a role in relieving the symptoms of asthma (Ryanna et al., 2009). In a study by Onishi et al. (2008), it was shown that Tregs (irrespective of their origin) interfere with the maturation of spleen derived and plasmacytoid dendritic cells (DC). They down regulate CD80/86 (but not CD40 and class II MHC) by attaching themselves and aggregating on splenic or plasmacytoid DCs. Such immature DCs are also called tolerogenic DCs and may play a role in regulating the response to allergies and asthma (Onishi et al., 2008). However, contrary to as mentioned in the review by Onishi et al. (2008), Ryanna et al. (2009) do not prescribe any role to TGF β or IL-10 in interaction to DCs or subsequent down regulation of CD80/86. Interestingly, Oertli et al. (2012), showed that naïve T cells were converted to Treg cells by IL-18 secreted from semi mature DC, thus forming a kind of loop where Tregs down regulate CD80/86 to form semi mature DCs which in turn further secrete IL-18 to convert naïve T cells to Tregs.

### Bringing *H. pylori* Effectors into the Picture

Even as the immunology of asthma is being understood despite its complexity, a role of microorganisms, especially *H. pylori*, in keeping with its inverse association with asthma is a topic of active research. Arnold et al. (2011) developed a mice model-based study where the hypothesis of *H. pylori* abrogating asthma was tested in both neonates and adults that were experimentally infected with *H. pylori* and exposed to alum-adjuvanted asthma allergens. Results showed that asthma was drastically reduced in neonatal mice infected with *H. pylori* but not in mice that were infected with *H. pylori* in adulthood. Also, the infected mice had higher counts of CD4^+^FoxP3^+^Treg cells and pulmonary infiltrating semi mature DC expressing low/intermediate (class II major histocompatibility Complex) MHCII. When the Tregs were purified from neonatally infected mice, it also conferred protection to uninfected mice. However, their study also showed that CagA had no role whatsoever in this immune response that protects against asthma and other allergies (Arnold et al., 2011). As mentioned above, in another follow-up study by the same group (Oertli et al., 2012), it was shown that naïve T cells were converted to Treg cells by IL-18 secreted from semi mature DC. This study also showed the importance of neonatal infection and explains why adults infected with *H. pylori* do not respond similar to the neonatally infected mice when exposed to allergens. This may be due to difference in IL-18 production in neonates and adults or may be due to barrier difference between neonatal gut and adult gut (Matsushima and Nagai, 2012). A role of IL-10 expressed in Treg cells has been implicated in persistent infection and protection from autoimmune disorders and allergies like asthma (Oertli et al., 2012).

## Hypothesis: How does *H. pylori* Modulate Immune Response?

As reported by Amedei et al. (2006), the apparent protection against asthma provided by the *H. pylori* infection relies on the redirection of typical Th2 responses toward a Th1 response. HP-NAP (*H. pylori* neutrophil-activating protein), which was found to be important for both immunity and pathogenesis (Satin et al., 2000; Cooksley et al., 2003), may play a role in this redirection of immune response. The experimental evidence provided includes increase in IFN-γ producing T-cells and decrease in IL-4 secreting cells, when HP-NAP was added to allergen-induced T-cell lines (Amedei et al., 2006). Both systemic and mucosal administration of HP-NAP was found effective in preventing allergic asthma (Codolo et al., 2008; D’Elios et al., 2009).

Given the vast numbers of microorganisms, both pathogenic and commensal, inhabiting the human body, a precise knowledge of the microbial effector molecule(s) involved in modulating Tregs is critical for enhancing our knowledge of the mechanisms involved in the development of immune tolerance. We propose that the direct interaction of HP-NAP with host Treg receptors results in the modulation of the host immune system to allow *H. pylori* persistence and at the same time suppression of the immune response to act against asthma inducing allergens. A HECT-type (Homologous to E6-associated protein C-Terminus-type) E3 ubiquitin ligase named Itch, has been implicated to regulate the conversion of Tregs to Th2 lymphocytes. Itch inhibits the expression of GATA3 (GATA-binding protein 3) and phosphorylation of STAT6 (signal-transducer and activator of transcription protein 6) thus inhibiting the expression of *Il4* gene that codes for cytokine IL-4. In a mouse model with a conditional Treg lineage-specific Itch deletion (*Itch^f/f^Foxp3^Cre^*), the Tregs show expression of GATA3 and activated STAT6 and hence IL-4 is produced which further prompts the naïve CD4^+^ T cells to initiate the Th2 differentiation program (Jin et al., 2013).

**FIGURE 1:**
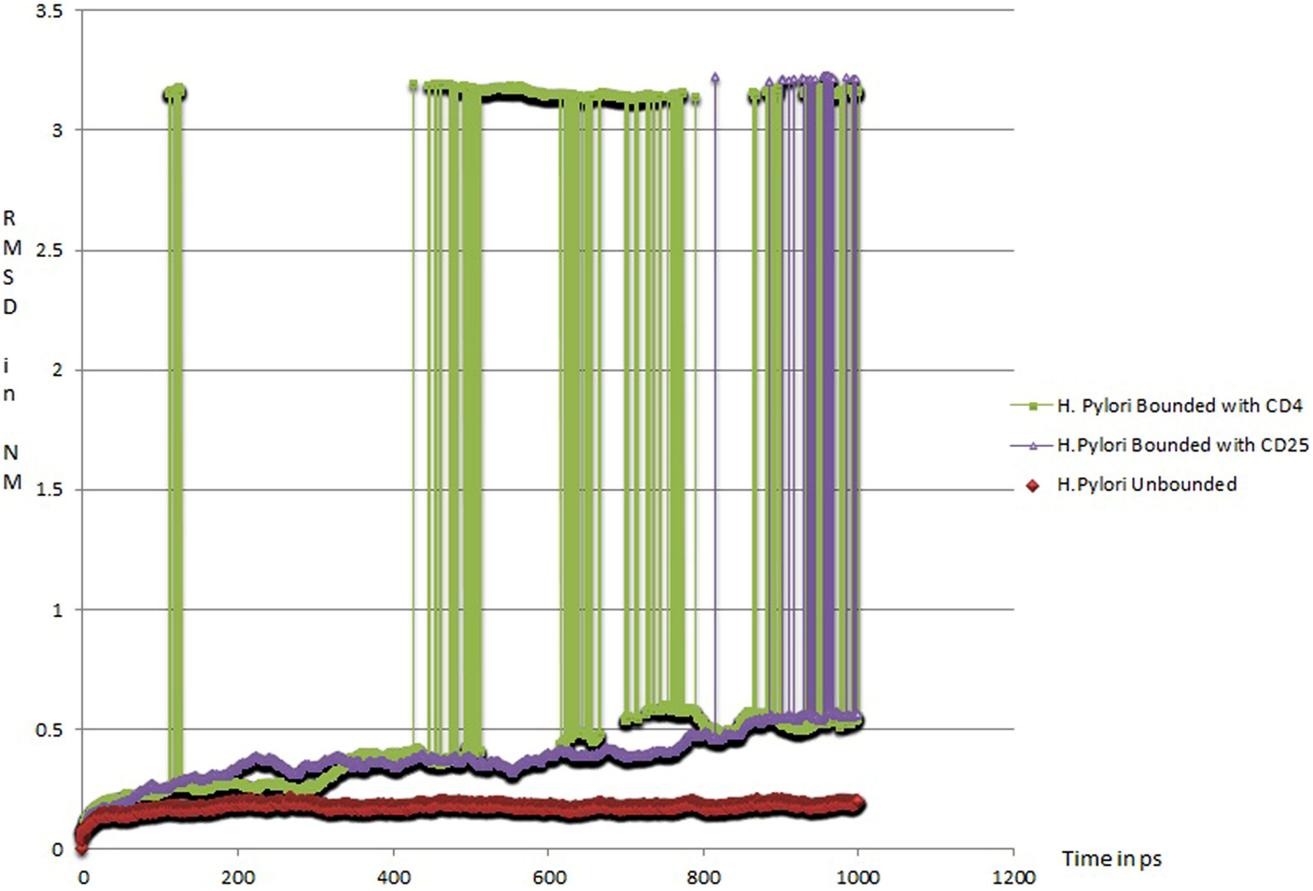
**Root Mean Square Deviation (RMSD) of protein complexes with respect to time (1 ns): (a) HP-NAP native structure (red in color; PDBID: 3T9J), (b) HP-NAP structure bounded with CD25 (violet in color), and (c) HP-NAP structure bounded with CD4 (green in color)**.

In order to determine likely candidate receptors on the Treg cell surface for HP-NAP, we initially narrowed our choices to CD4 and CD25. The former is ubiquitously expressed on T helper cells and Tregs, while the latter is expressed at high levels on Tregs in humans (Baecher-Allan et al., 2001), and constitutes the alpha chain of the heterotrimeric IL-2 receptor. To further estimate the relative strength of HP-NAP with these receptors, we have undertaken protein dynamics simulations of HP-NAP (PDB ID: 3T9J) complexed with CD4 (PDB ID: 1CDJ) or CD25 (modified from PDB ID: 1Z92; see Supplementary Material). The simulations were carried out over a time period of 1000 ps (1 ns). The CD25-HP-NAP complex demonstrated a greater stability in the form of root mean square deviation (RMSD) value plotted against time as compared to CD4-HP-NAP complex. The CD4-HP-NAP complex aligns itself in a more vertical position and subsequently loses contact with the active site at around 50 ps and remains unstable throughout the simulation period, whereas the CD25-HP-NAP complex orients itself in the original binding conformation and remains stable for the rest of the simulation (data unpublished, see **Figure 1**). This supports our hypothesis that HP-NAP interacts with Treg cells using the CD25 receptor and is supportive of the claim by Corthay (2009) that Tregs are activated and elicit their immunosuppressive activity in an antigen-specific manner.

**FIGURE 2:**
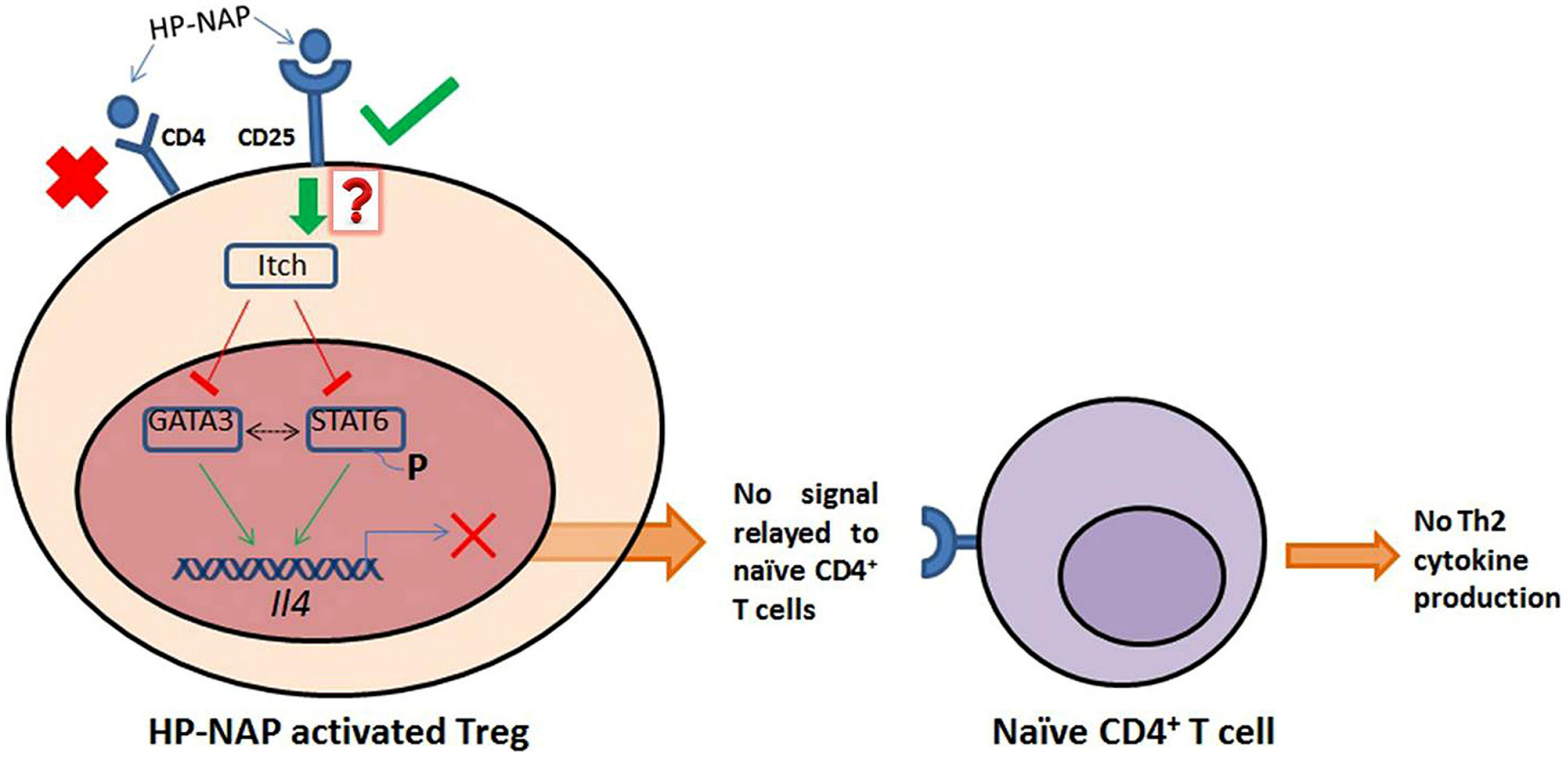
**HP-NAP binds stably with CD25 receptor present on the surface of Treg cells**. The binding between HP-NAP and CD4 receptor is found to be unstable and therefore it is not suppose to play any role in Itch activation. The activated Itch plays important role in suppressing the expression of GATA3 and phosphorylation of STAT6, which prevents the transcription of *Il4* gene. Ultimately, in the absence of IL-4 production, no message for Th2 cytokines production is relayed to naïve CD4^+^ T cells. In this manner, the presence of *Helicobacter pylori* in the host shifts the Th2 response toward Th1 response. Red “X” indicates non-feasible attachments or reactions; green arrows indicate positive associations, and red lines indicate negative effects. Dotted black line indicates the unavailability of sure evidence. The red question mark indicates unknown mechanism. Adapted from Figure 1 in Chen (2013), with permission.

Thus, we propose HP-NAP interacts with Regulatory T cells through the CD25 surface receptor and then the signal is relayed inside the Tregs (by an as yet unknown mechanism) which leads to expression and activation of Itch (see **Figure 2**). The activated ubiquitin ligase suppresses the expression of GATA3 and phosphorylation of STAT6, therefore preventing *Il4* gene transcription and IL-4 production. In this condition naïve CD4^+^ T cells will not be receiving the signal for further production of any of the Th2 cytokines (IL-4, IL-5, and IL-13).

## Concluding Remarks

Along with numerous positive aspects, improvements in sanitation and usage of antibiotics also bring about significant changes in our indigenous microbiota. As a part of this change, the incidence of *H. pylori* infection in the human host (among children) has decreased. The unintended consequence of of improved hygiene and medicine is probably an increasing incidence of allergy in the pediatric population. In the specific case of *H. pylori*, its persistence in the human stomach alters the Th1/Th2 cytokine balance and ultimately shifts the Th2 response toward Th1 via Treg modulaton. Computational analysis indicates a stable interaction of HP-NAP with CD25 (IL-2Rα) expressed exclusively on Tregs. By contrast, no such stable interaction is predicted for HP-NAP with CD4, expressed on both T helper cells and Tregs. Therefore, our simulations of HP-NAP interactions with Treg cell surface molecules provide valuable insights and generate testable hypotheses to develop a model of the antigen-specific immunosuppressive activity of Tregs.

## Conflict of Interest Statement

The authors declare that the research was conducted in the absence of any commercial or financial relationships that could be construed as a potential conflict of interest.
