# Supplementary material for: Helicobacter pylori neutrophil-activating protein: a potential Treg modulator suppressing allergic asthma?
Source: Front Microbiol. 2015 Jun 1;6:493. doi: 10.3389/fmicb.2015.00493 (PMC4450655; doi:10.3389/fmicb.2015.00493)
Supplement: Supplementary file 1 [file Data_Sheet_1.PDF]

Supplementary Material

Helicobacter pylori neutrophil-activating protein: A potential T_{reg} modulator suppressing allergic asthma?

Sehrawat A[1,*], Sinha S[1], Saxena A[1]

[1]Department of Biotechnology, TERI University, New Delhi, India.

*** Correspondence:** Anjna Sehrawat, PhD Scholar, Department of Biotechnology, TERI University, New Delhi-110070, India. adhanda.biotech@gmail.com

1. Supplementary Data

The protein structure for H pylori was docked with that of CD4 and CD25 respectively using the clus pro server. The fully automated docking and discrimination server ClusPro can be found at http://structure.bu.edu.

Clus pro utilizes the fast algorithm for filtering docked conformations with good surface complementarity and ranking them based on their clustering program. The models generated are ranked as per the cluster size, the protein is rotated with 70,000 rotations, for each rotation the protein is translated in x, y, z relative to the receptor in the grid, thus the translation with best score is chosen from each rotation. Out of the 70,000 rotation, the top 1000 rotations with lowest score have been selected. Clustering is then used to smooth the local minima and to select the ones with the broadest energy wells, a property associated with the free energy at the binding site - 9Å C- alpha rmsd radius,i.e. Position with most neighbours in 9 Angstrom as it becomes the cluster centre and its neighbours the members of the cluster. (table1).

The selected protein complexes were subjected to Molecular Dynamics Simulation for a time period of 1000ps (1ns), using the software GROMACS 5.0

2. Supplementary Figures and Tables

2.1. Supplementary Tables

Clusters of *H. pylori* with CD25				Clusters of *H. pylori* with CD4			
Cluster	Members	Representative	Weighted Score (kcal/mol)	Cluster	Members	Representative	Weighted Score (kcal/mol)
0	161	Center	-803.6	0	161	Center	-577.3
0	161	Lowest Energy	-953.5	0	161	Lowest Energy	-711.4
1	96	Center	-772.9	1	97	Center	-616.8

1	96	Lowest Energy	-859.9	1	97	Lowest Energy	-668.1
2	91	Center	-864.6	2	83	Center	-703.3
2	91	Lowest Energy	-934.8	2	83	Lowest Energy	-713.7
3	82	Center	-913.5	3	55	Center	-571.5
3	82	Lowest Energy	-915.6	3	55	Lowest Energy	-632.8
4	78	Center	-788.2	4	53	Center	-630.6
4	78	Lowest Energy	-946.4	4	53	Lowest Energy	-737.3
5	54	Center	-727.3	5	46	Center	-575.3
5	54	Lowest Energy	-866.4	5	46	Lowest Energy	-660.8
6	51	Center	-789.5	6	42	Center	-591.8
6	51	Lowest Energy	-925.1	6	42	Lowest Energy	-641
7	48	Center	-724.9	7	41	Center	-577.1
7	48	Lowest Energy	-835.5	7	41	Lowest Energy	-605.6
8	41	Center	-792.4	8	39	Center	-577
8	41	Lowest Energy	-811.5	8	39	Lowest Energy	-605.2
9	40	Center	-796.1	9	30	Center	-609.8
9	40	Lowest Energy	-812.7	9	30	Lowest Energy	-678
10	36	Center	-717.5	10	30	Center	-606.8
10	36	Lowest Energy	-867.7	10	30	Lowest Energy	-685.3
11	29	Center	-742.8	11	30	Center	-624.6
11	29	Lowest Energy	-803.7	11	30	Lowest Energy	-635.8
12	28	Center	-736	12	29	Center	-593.4
12	28	Lowest Energy	-797.3	12	29	Lowest Energy	-626.6

Table S1: Protein models (*H. pylori* with CD25 and CD4) generated through Clus Pro demonstrates docking energy in kcal/mol and have been ranked according to the cluster size.

2.2. Supplementary Figures

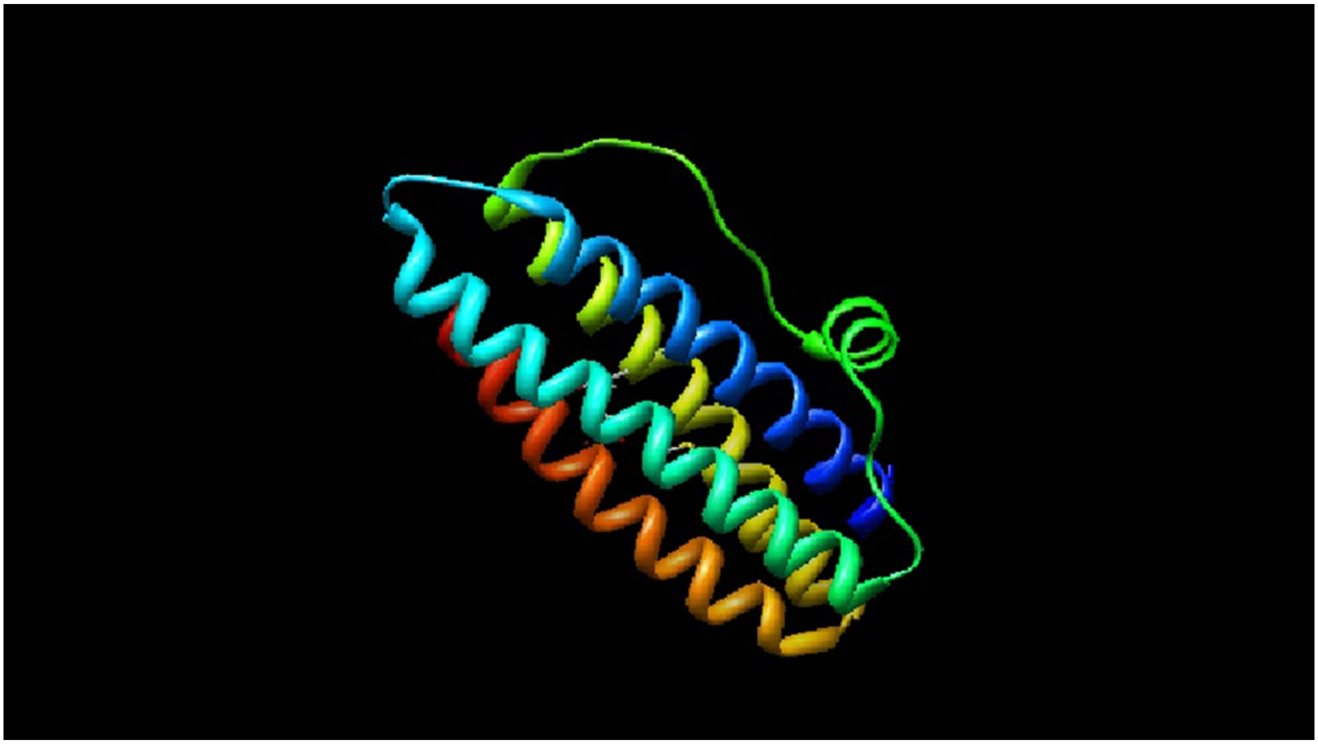

Figure S1: HP-NAP (PDBID: 3T9J)

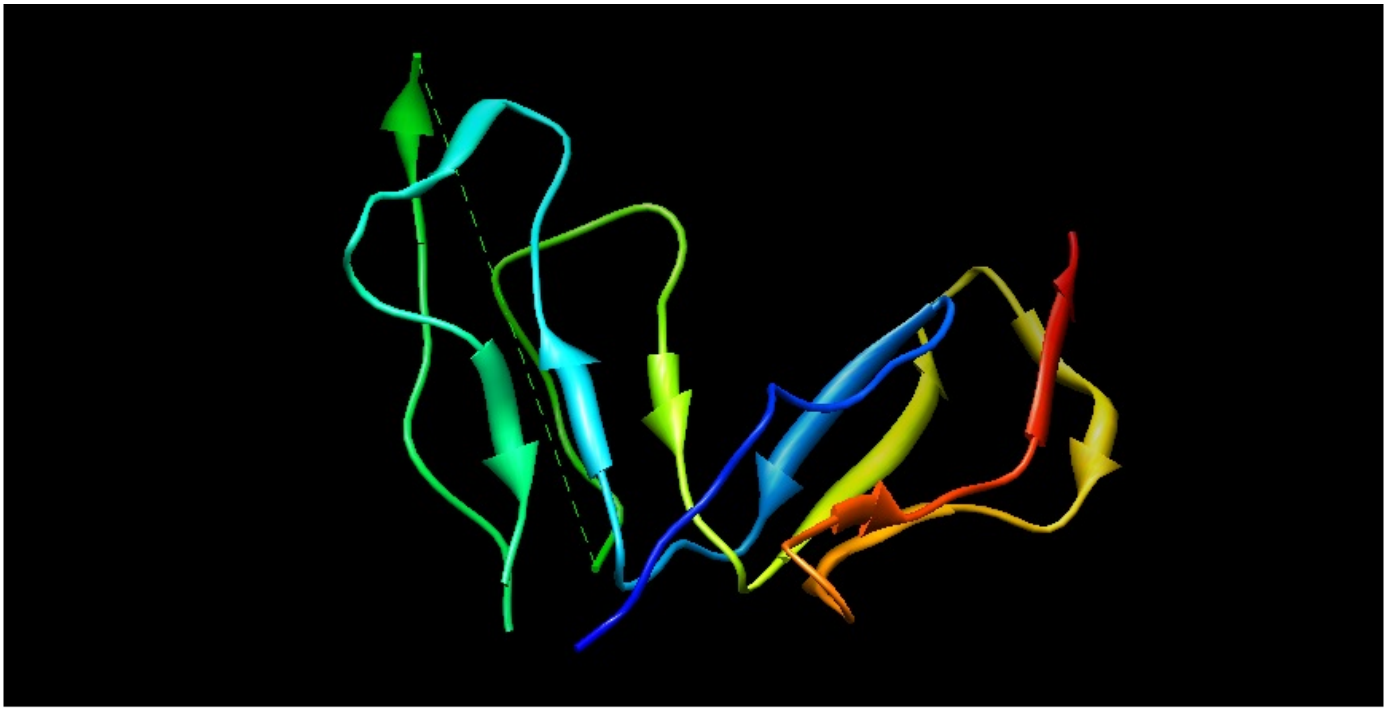

Figure S2: The crystal structure of CD25 modified from crystal structure showing IL-2 complexed with CD25 (PDBID: 1Z92).

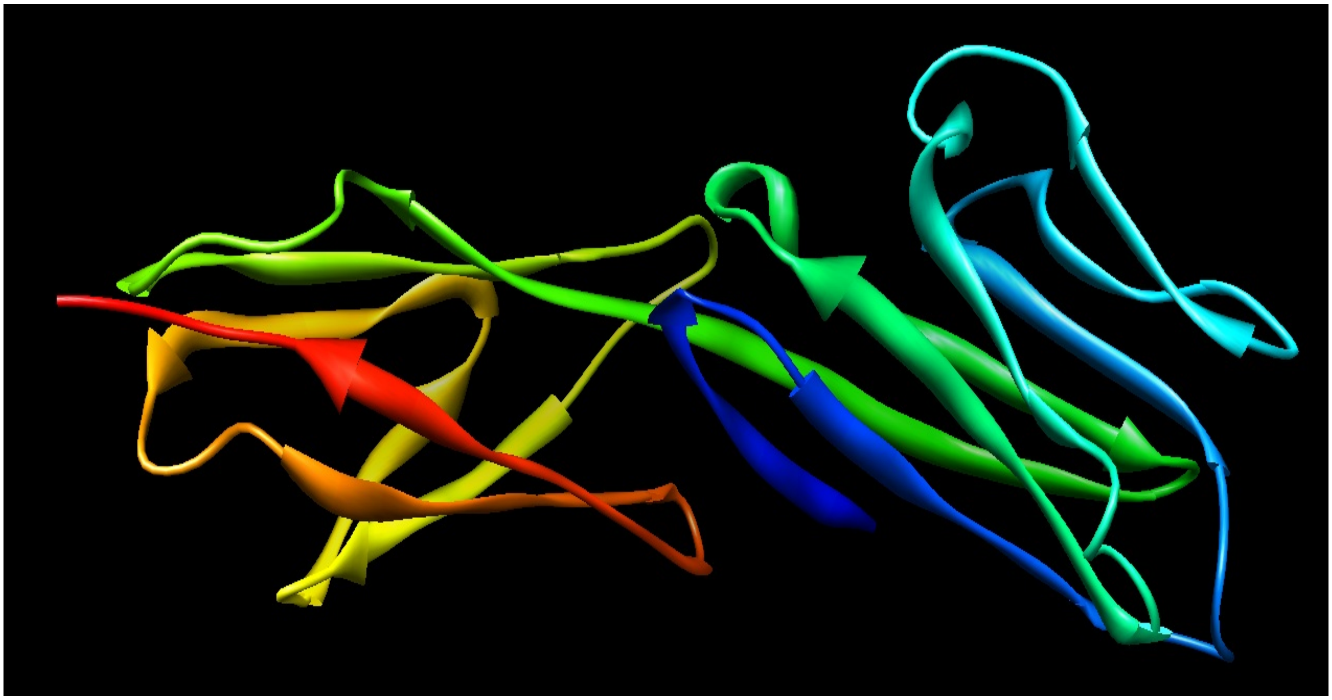

Figure S3: The crystal structure of CD4 (PDB ID: 1CDJ).

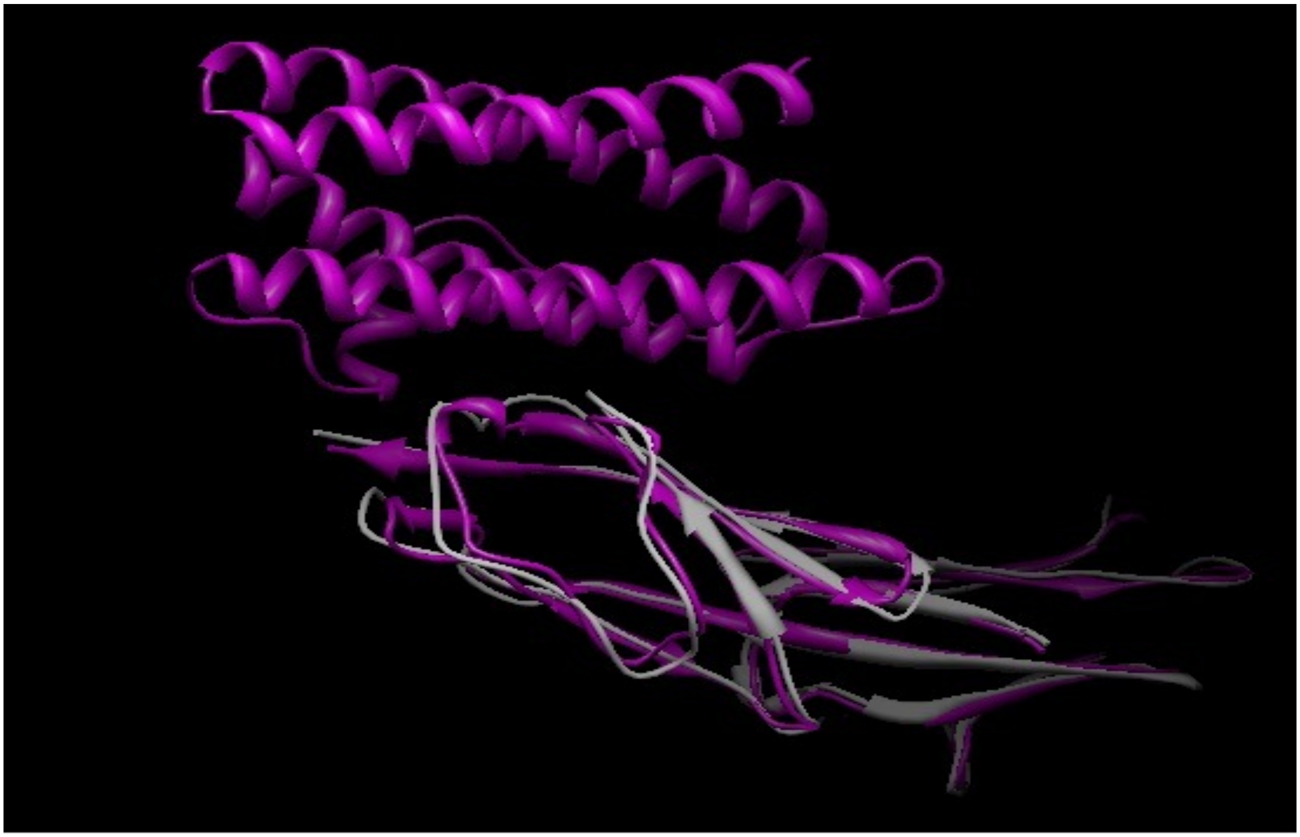

Figure S4: Post Docking structure alignment of H.pylori-CD25 structure complex with that of native CD4 structure (PDB ID: 1CDJ).

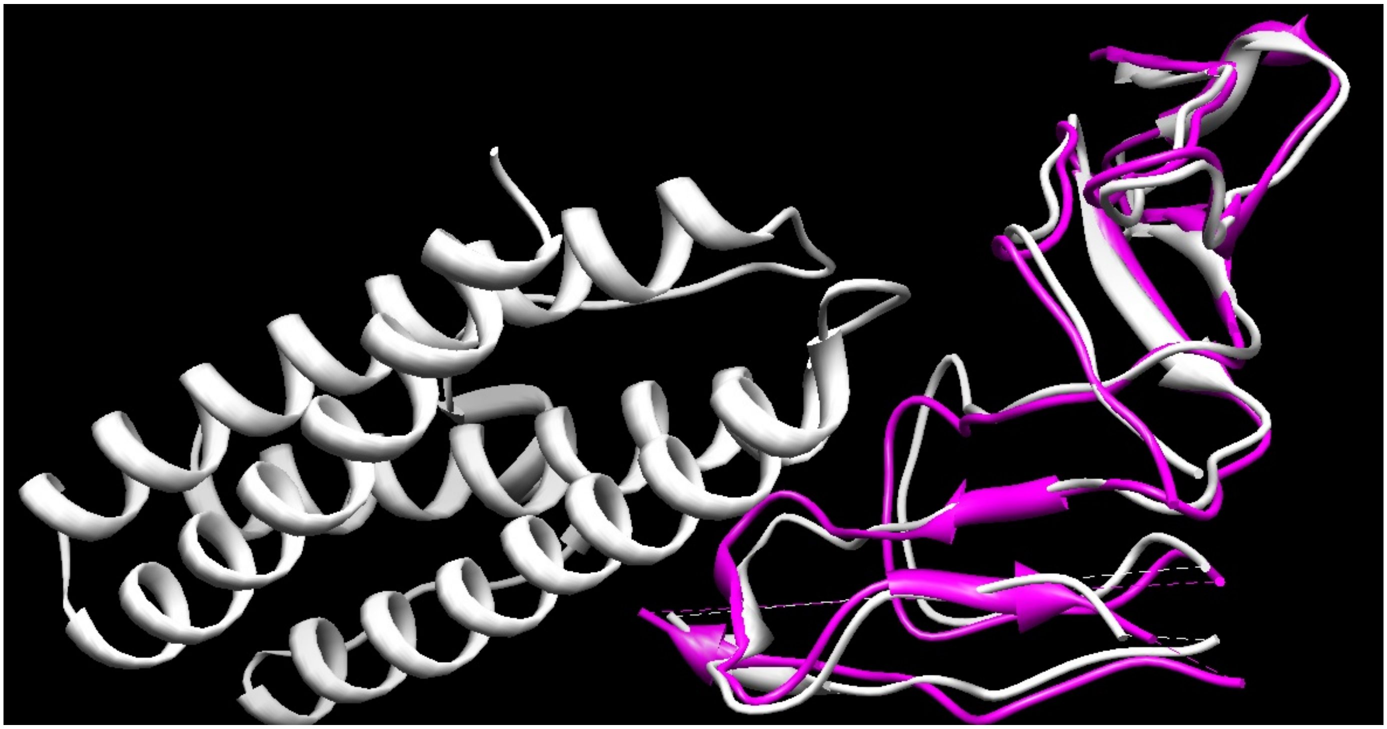

Figure S5: Post Docking structure alignment of H.pylori-CD4 structure complex with that of native CD25 structure (PDB ID: 1Z92).

3. References[1]

Ozakov, D., Beglov, D., Bohnuud, T., Mottarella, S., Xia, B., Hall, D.R., Vajda, S. (2013). How good is automated protein docking? *Proteins: Structure, Function, and Bioinformatics*, 81:2159–2166.

Kozakov, D., Brenke, R., Comeau, S.R., Vajda, S. (2006). PIPER: An FFT-based protein docking program with pairwise potentials.*Proteins: Structure, Function, and Bioinformatics*, 5:392–406.

Comeau, S.R., Gatchell, D.W., Vajda, S., Camacho, C.J. (2004) ClusPro: an automated docking and discrimination method for the prediction of protein complexes. *Bioinformatics*, 20:45-50.

Comeau, S.R., Gatchell, D.W., Vajda, S., Camacho, C.J. (2004) ClusPro: a fully automated algorithm for protein-protein docking.*Nucleic Acids Research*, 32:96-99.

Hess, B., Kutzner, C., van der Spoel, D.,Lindahl, E. (2008). GROMACS 4: Algorithms for highly efficient, load-balanced, and scalable molecular simulation. J. Chem. Theory Comput, 4:435-447.

Van der Spoel, D., Lindahl, E., Hess, B., Groenhof, G., Mark, A.E., Berendsen, H.J.C.(2005)GROMACS: Fast, Flexible and Free. J. Comp. Chem, 26: 1701-1719.

[1] Provide the doi when available, and ALL complete author names.
